# Health-related quality of life in French adults with X-linked hypophosphatemia: real-world data from the International XLH Registry

**DOI:** 10.1093/jbmrpl/ziag050

**Published:** 2026-03-25

**Authors:** Karine Briot, Sandrine Lemoine, Bernard Cortet, Paulina Szafors, Nadia Mehsen, Guillaume Couture, Kerry Sandilands, Angela J Rylands, Haruka Ishii, Annabel Bowden, Jennifer E Dent, Peter Kamenicky

**Affiliations:** Rheumatology Department, Cochin Hospital, Université Paris Cité and Université Sorbonne Paris Nord, Inserm, INRAE, Center for Research in epidemiology and Statistics (CRESS), F-75004 Paris, France; Centre de Référence des Maladies Rares du Calcium et du Phosphore, Centre de Référence des Maladies Rénales Rares, Filières OSCAR et ORKID, Bron 69003; Filières Européennes Bond et ERKNet, Nephrology and Renal Physiology Department, 69003 Lyon, France; Faculté de Médecine Lyon Est, Hôpital Edouard Herriot, 69003 Lyon, France; Faculté de Médecine Lyon Est, Université Claude Bernard Lyon 1, 69100 Villeurbanne, France; Rheumatology Department and ULR 4490 (MabLab), University-Hospital of Lille, 5900 Lille, France; Rheumatology Department, Hopital Lapeyronie, 34090 Montpellier, France; Department of Rheumatology, Groupe Hospitalier Pellegrin, 33000 Bordeaux, France; Rheumatology Department, Centre Hospitalier Universitaire de Toulouse, 31300 Toulouse, France; Health Economics Outcomes Research, Kyowa Kirin International, Marlow SL7 1HZ, United Kingdom; Health Economics Outcomes Research, Kyowa Kirin International, Marlow SL7 1HZ, United Kingdom; Health Economics Outcomes Research, Kyowa Kirin Co., Ltd., 100-0004 Tokyo, Japan; Patient Centred Outcomes, Spire Outcomes Limited, London N1 7GU, United Kingdom; Analytics, Spire Outcomes Limited, London N1 7GU, United Kingdom; Adult Enocrinology Service, Centre de Référence des Maladies Rares du Métabolisme du Calcium et du Phosphate, Filière OSCAR, Assistance Publique-Hôpitaux de Paris (AP-HP), Hôpital de Bicêtre, Service d'Endocrinologie et des Maladies de la Reproduction, 94270 Le Kremlin-Bicêtre, France; Physiologie et Physiopathologie Endocriniennes, Université Paris-Saclay, Inserm, 91190 Gif-sur-Yvette, France

**Keywords:** X-linked hypophosphatemia (XLH), patient-reported outcomes, health-related quality of life (HRQL), burosumab, registry, real-world data

## Abstract

X-linked hypophosphatemia (XLH) is a rare genetic condition in which excess fibroblast growth factor 23 causes renal phosphate wasting, leading to skeletal morbidities. Patients experience musculoskeletal pain, stiffness, and fatigue, with impaired physical function and health-related quality of life (HRQL). Burosumab has been available in France for the treatment of XLH since 2021; European treatment guidelines suggest use in adults with pseudofractures or with insufficient response and/or intolerance to oral phosphate supplements and active vitamin D. The International XLH Registry is collecting long-term observational data in a real-world setting from patients with XLH. Here, we report data from French adults from their first completion of the Short-Form 36 (SF-36) version 2 health survey, a measure of health-related quality of life. The analysis used data from 123 adults who completed the survey at least once (73% women; mean age at completion 42.5 yr [SD 13.4]; mean age at diagnosis 8.8 [12.6] yr). Group mean T-scores in the current International XLH Registry sample were <47 on all SF-36 scale and summary scores, indicating impaired functioning. Physical and mental component summary scores were similar (43.0 [SD 9.1] vs 43.4 [11.3]). Worse physical component summary scores were significantly related to older age (*p* = .032), not working (excluding students) (*p* < .001), history of fracture (*p* < .001), lower-extremity fracture (*p* = .010), surgery as an adult (*p* < .001), current burosumab treatment (*p* = .006), and previous burosumab treatment (*p* = .002), possibly because treatment guidelines direct use to patients with more severe disease. Lower mental component summary scores were related to younger age (*p* = .010). Physical summary scores were better in the current analysis than in adults with other chronic musculoskeletal conditions, mental summary scores were worse. Further exploration of the relationships between modifiable influences on HRQL is warranted, including the impact of earlier intervention with burosumab on physical symptoms, notably fractures, and HRQL.

## Graphical Abstract

**Graphical Abstract:**
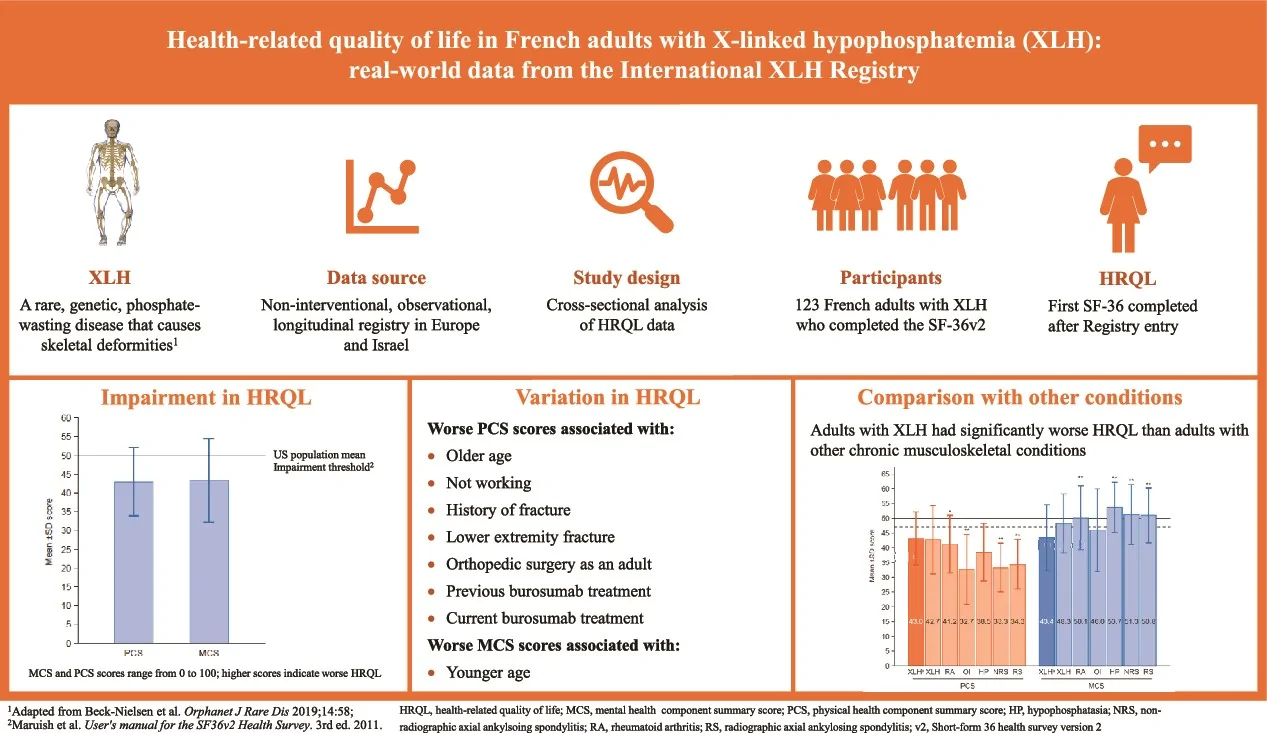

## Introduction

X-linked hypophosphatemia (XLH) is a rare, genetic disorder, characterized by renal phosphate wasting due to excess activity of fibroblast growth factor 23 (FGF23), caused by inactivating mutations in the *PHEX* (phosphate-regulating endopeptidase homologue, X-linked) gene. Excess FGF23 activity also impairs 1,25(OH)_2_D_3_ production, compromising intestinal phosphate absorption.^1^ Chronic hypophosphatemia during childhood compromises skeletal development and growth.^1–3^ Skeletal complications may continue to accumulate if hypophosphatemia persists into adulthood^4^^,^^5^; these include fractures, pseudofractures, early onset osteoarthritis with osteophytes, enthesopathy, and spinal stenosis. Patients with XLH may also have reduced muscle strength and function,^6–8^ musculoskeletal pain, and stiffness, and physical function may be impaired.^5^

Health-related quality of life (HRQL) is an intrinsically multidimensional patient-reported outcome; it is defined by the European Medicines Agency as the patient’s subjective perception of the impact of their disease and its treatment(s) on their daily life, physical, psychological and social functioning, and wellbeing.^9^ The symptom burden of XLH, particularly pain, stiffness, and fatigue, has been reported to affect HRQL, compromising psychological and emotional wellbeing, physical activity, and activities of daily living^10–14^ and work productivity.^15^ Real-world studies using the Short-Form 36 Health Survey (SF-36) have reported impaired HRQL in adults with XLH^11^^,^^16–21^; however, the impact of patient characteristics, symptoms, and treatment on HRQL in adults with XLH has yet to be explored. Improvement of HRQL has been identified as a long-term goal in the treatment of XLH.^22^

Evidence-based treatment guidelines published in 2025 recommend treatment for adults with significant symptoms and manifestations of XLH and those undergoing planned orthopedic or dental implant surgery.^23^ The guidelines recommend oral phosphate supplements and active vitamin D for adults with biochemical and/or clinical signs of osteomalacia, musculoskeletal pain, or stiffness. European treatment guidelines suggest burosumab for the treatment of adults with pseudofractures or insufficient musculoskeletal response to oral phosphate supplements and active vitamin D, and for symptomatic patients who do not tolerate this treatment or experience adverse effects (eg, Progressive nephrocalcinosis, Kidney stones, Hypercalcemic hyperparathyroidism, Persistent gastrointestinal discomfort, and Diarrhea). Burosumab has been reimbursed for patients with severe XLH in France since 2021. It is a fully human monoclonal antibody that directly targets the underlying cause of XLH by binding to and inhibiting FGF23. In clinical studies it has been shown to increase serum phosphate concentration, with improvements in biomarkers of bone turnover, osteomalacia-related histomorphometric measures, bone healing, and skeletal complications of XLH.^24^^,^^25^ In phase 3 clinical trials, adults treated with burosumab also had statistically significant improvements from baseline in patient-reported stiffness, pain, fatigue, and/or physical activity at 24, 48, and/or 96 wk of treatment, and clinically meaningful improvement was also seen in several of these measures.^25–27^ These improvements were maintained with long-term treatment (up to 144 wk).^28^

The International XLH Registry is a non-interventional observational program that was established to address a gap in knowledge on the natural history of XLH, and to evaluate the impact of treatment and other medical interventions on patient outcomes through long-term collection of data in a real-world setting^29^; it is enrolling children and adults with XLH.^30^

The objectives of the current analysis were to describe the HRQL of adults with XLH in France using data from the International XLH Registry, to examine variation in HRQL according to patient, XLH, and treatment characteristics, and to compare HRQL scores in adults with XLH with scores from adults with other chronic musculoskeletal conditions.

## Materials and methods

### Study data

The International XLH Registry was established across sites in Europe and Israel in August 2017, as a 10-yr observational real-world data collection program.^29^^,^^30^ The XLH Registry includes patients of any age who have a clinical presentation or radiological, biochemical, genetic, or family mapping investigation results that support a diagnosis of XLH at the enrolment visit. Patients were enrolled regardless of treatment for XLH (phosphate supplements and/or active vitamin D, burosumab, or currently untreated); however, patients involved in any interventional clinical trial were not eligible for enrolment until completion of that trial.

The XLH Registry is run following the recommendations from the Declaration of Helsinki and has received ethical approval at national, regional, and site levels as required in each participating country. Patient data held within the registry is kept in accordance with the EU General Data Protection Regulations on the processing of personal data and the protection of privacy in the electronic communication sector (2016/679/EU). Adult patients gave written informed consent for inclusion in the International XLH Registry.

The International XLH Registry does not specify follow-up requirements. Retrospective data on general medical history was collected for a recommended 6 yr prior to Registry entry. XLH treatments, laboratory data, and HRQL were captured during prospective patient visits. Following a protocol amendment in October 2021, adults enrolled in the International XLH Registry were asked to complete the SF-36 version 2 at clinic visits (completion was recommended but optional).

### Analyses

The current analysis used data through to October, 31 2023 from adults (age ≥18 yr at Registry entry) who enrolled in the International XLH Registry at French centers between December 19, 2018 and July 28, 2022. Patients with XLH who had a non-*PHEX* mutation confirmed by genetic test were not eligible for the current analysis.

### Patient characteristics

Patient characteristics, including demographics, clinical history, and treatment history, are reported for all adults at baseline (defined as the closest recorded data entry within 1 yr of giving informed consent at Registry entry) and, for those who completed the SF-36, at baseline and at first SF-36 completion. Demographic variables included age, age category, sex, and employment status. Medical history variables included height, weight, and BMI Z-scores, *PHEX* mutation status, family history of XLH, age at XLH diagnosis, time from XLH diagnosis to registry entry, serum bone biochemistry (values and normality of phosphate, alkaline phosphatase (ALP), 1,25(OH)_2_D_3_, and parathyroid hormone (PTH)), number and type of lower-limb deformity, fracture history, and surgical history. Treatment variables included current and previous treatment for XLH, time on burosumab, dose of burosumab, and age at start of burosumab treatment.

### HRQL

The SF-36 evaluates HRQL over the previous month based on 8 scales: Physical functioning, Role limitations due to physical health (Role—Physical), Bodily pain, General health perceptions, Vitality, Social functioning, role limitations due to emotional problems (Role—Emotional), and ental health. The 8 scale scores are used to calculate the Physical and Mental Component Summary (PCS and MCS) scores and a total score. A linguistically validated version of the SF-36 in French was used.

Data collected at the first SF-36 completion after registry entry were analyzed, regardless of the time since registry entry. The SF-36 was scored using QualityMetric Health Outcomes Scoring Software.^31^ All scale, summary, and total scores range from 0 to 100, with higher scores indicating better HRQL/fewer problems or symptoms; a scale or summary score <47 indicates impaired functioning.^31^ The norm-based T-scores generated by the software enable comparisons across scales and component summary scores, facilitating interpretation in relation to the distribution of scores in the 2009 US general population.^31^ Missing data were handled according to the scoring guide: if the SF-36 was not completed, the score was set to missing; at least 50% of the items had to be present for a scale score to be calculated. HRQL scores at first SF-36 completion are reported for all patients and by age category. For the descriptive analysis of item-level data, response options were collapsed into 3 categories to allow comparison across items where there were different numbers of responses.

### Statistical analysis

Continuous variables are summarized by number of subjects, mean, SD, and range, and the categorical variables are reported as number and percentage of patients.

Variation in SF-36 scores by demographic, medical history, and treatment history variables was assessed using Student’s *t*-test/Wilcoxon rank-sum test for 2 categories and 1-way/Kruskall–Wallis analysis of variance (ANOVA) for more than 2 categories, depending on the normality of the data distribution. Pearson’s or Spearman’s rank correlation coefficients were determined for continuous data depending on the normality of the data distribution. Not all data were available at the time of SF-36 completion so baseline data were used where plausible (eg, employment status). The burosumab dose (mg/kg) at SF-36 completion was determined using baseline body weight values, as body weight is not expected to change notably from enrolment to SF-36 completion.

Variables for which there were insufficient data at the time of SF-36 completion (eg, serum bone biochemistry) were excluded from the bivariate analysis.

Student’s *t*-tests were used to compare the SF-36 scores from the current analysis with reported values for US adults with XLH^32^ and values reported for adult populations with other chronic musculoskeletal conditions: rheumatoid arthritis,^33^ osteogenesis imperfecta (OI), hypophosphatasia,^32^ and radiographic and non-radiographic axial spondyloarthritis.^34^

Statistical significance testing was 2-sided at the 5% level. Multiple comparisons were not accounted for because all analyses were performed in an exploratory manner. Analyses were performed using SAS 9.4.

## Results

### Study population

The analysis used data from 181 adults with XLH who were enrolled in the International XLH Registry at French centers between December 19, 2018 and July 28, 2022. Of these, 123 (68%) had completed the SF-36 at least once and were included in the current analysis. The mean (SD) time from baseline to first SF-36 completion was 18.6 (13.8) mo (range 0.1-46.8). Table 1 presents demographic and clinical history at baseline for all adults, and at baseline and at first SF-36 completion for those who completed the SF-36. Baseline demographic and clinical history characteristics of patients who completed the SF-36 were mostly similar to those for the whole adult population but with some minor differences in age distribution and employment status.

**Table 1:** Demographics and clinical history at baseline and first SF-36 completion.

**Variable**		**SF-36 completers (*n* = 123)**		**All adults at baseline (*n* = 181)**
		**At baseline**	**At first SF-36 completion**	
**Sex, *n* (%)**	Female	90 (73.2)		134 (74.0)
	Male	33 (26.8)		47 (26.0)
**Age (yr)**	Mean ± SD	41.0 ± 13.4	42.5 ± 13.4	40.3 ± 15.0
	Range	18.0-79.0	18.0-79.0	18.0-79.0
**Age category (yr), *n* (%)**	18 to <30	25 (20.3)	23 (18.7)	49 (27.1)
	30 to <50	62 (50.4)	60 (48.8)	79 (43.6)
	≥50	36 (29.3)	40 (32.5)	53 (29.3)
**Employment status, *n* (%)**	Student	5 (4.1)	NR	16 (8.8)
	Employed/self-employed	51 (41.5)	NR	70 (38.7)
	Not employed	6 (4.9)	NR	10 (5.5)
	Retired/disability/homemaker	11 (8.9)	NR	14 (7.7)
	Unknown/NR	50 (40.7)	NR	71 (39.2)
**Height (cm) Z-score^a^**	*n*	57	NR	87
	Mean ± SD	−2.2 ± 1.6		−2.1 ± 1.5
	Range	−5.9 to 2.0		−5.9 to 2.0
**Weight (kg) Z-score^a^**	*n*	71	NR	105
	Mean ± SD	−0.2 ± 1.0		−0.4 ± 0.9
	Range	−1.8 to 3.3		−2.0 to 3.3
**BMI (kg/m^2^) Z-score^a^**	*n*	53	NR	82
	Mean ± SD	0.6 ± 1.3		0.5 ± 1.2
	Range	−1.1 to 4.3		−1.1 to 4.3
**Age at diagnosis (yr)**	*n*	113		166
	Mean ± SD	8.8 ± 12.6		9.5 ± 14.9
	Range	0.0-50.0		0.0-76.3
**Time since XLH diagnosis (yr)**	*n*	113	113	166
	Mean ± SD	32.3 ± 15.4	33.8 ± 15.3	30.6 ± 15.7
	Range	3.6-75.9	4.0-76.0	0.4-75.9
**Diagnosis confirmed by *PHEX* mutation test, *n* (%)**	Yes	99 (80.5)		141 (77.9)
	No	24 (19.5)		40 (22.1)
**Family history, *n* (%)**	*n*	69	NR	101
	Yes	47 (68.1)	NR	68 (67.3)
	No	22 (31.9)	NR	33 (32.7)
**Family history, parents affected, *n* (%)**	*n*	69	NR	101
	Mother only	35 (50.7)	NR	48 (47.5)
	Father only	12 (17.4)	NR	20 (19.8)
	Both parents	0	NR	0
	Neither parent	22 (31.9)	NR	33 (32.7)
**Clinical history (yes), *n* (%)**	Club foot deformity	0	0	0
	Craniosynostosis	2 (1.6)	2 (1.6)	2 (1.1)
	Enthesopathy	41 (33.3)	42 (34.1)	50 (27.6)
	Excessive cavities	19 (15.4)	19 (15.4)	27 (14.9)
	Genu valgum	21 (17.1)	21 (17.1)	32 (17.7)
	Genu varum	34 (27.6)	35 (28.5)	49 (27.1)
	Intoeing	3 (2.4)	3 (2.4)	5 (2.8)
	Nephrocalcinosis	10 (8.1)	10 (8.1)	20 (11.0)
	Osteoarthritis	38 (30.9)	38 (30.9)	49 (27.1)
	Osteophytes	8 (6.5)	8 (6.5)	15 (8.3)
	Tibial torsion	0	0	0
	Tooth abscess	63 (51.2)	63 (51.2)	87 (48.1)
	Windswept deformity	0	0	0
**Number of categories for lower limb deformities, *n* (%)**	0	63 (51.2)	63 (51.2)	94 (51.9)
	1	45 (36.6)	45 (36.6)	64 (35.4)
	2	12 (9.8)	12 (9.8)	19 (10.5)
	3	2 (1.6)	3 (2.4)	3 (1.7)
	>3	1 (0.8)	0	1 (0.6)
**Fracture history (yes), *n* (%)**	Lower extremities	22 (17.9)	22 (17.9)	30 (16.6)
	Upper extremities	5 (4.1)	5 (4.1)	8 (4.4)
	Trunk	3 (2.4)	3 (2.4)	4 (2.2)
	Any fracture	26 (21.1)	26 (21.1)	37 (20.4)
**Surgical history (yes), *n* (%)**	Ankle replacement	0	0	0
	Craniotomy/craniectomy	3 (2.4)	3 (2.4)	3 (1.7)
	External fixation	0	0	0
	Fracture fixation with plates/screws	5 (4.1)	5 (4.1)	9 (5.0)
	Fracture fixation with intramedullary nail/rod	0	0	0
	Hip replacement	7 (5.7)	7 (5.7)	9 (5.0)
	Knee replacement	5 (4.1)	5 (4.1)	5 (2.8)
	Leg lengthening	0	0	1 (0.6)
	Osteotomy	74 (60.2)	74 (60.2)	101 (55.8)
	Stapling of growth plates	5 (4.1)	5 (4.1)	8 (4.4)
	Tibial torsion	0	0	0
	Any	79 (64.2)	79 (64.2)	109 (60.2)
**Time of surgery (yes), *n* (%)**	Adult	33 (26.8)	33 (26.8)	40 (22.1)
	Pediatric	57 (46.3)	57 (46.3)	78 (43.1)

a Z-scores based on the French adult population from the 2020 Obepi-Roche Study by the Ligue Contre l’Obesite.^40^

Abbreviations: NR, not reported; SF-36, Short-Form 36 Health Survey.

At SF-36 completion, the mean age was 42.5 yr (SD 13.4) and 73% of patients were female. The mean age at XLH diagnosis was 8.8 (SD 12.6) yr and the mean time since diagnosis was 33.8 (SD 15.3) yr. Most patients (81%) had a confirmed *PHEX* mutation and 68% had a family history of XLH. In terms of clinical history (Table 1), at SF-36 completion approximately half of patients had lower-limb deformities in at least one category (ie, club foot deformity, genu valgum, genu varum, intoeing, tibial torsion, or windswept deformity); of these, most had deformities in one category only (37%). About one-third of patients had osteoarthritis (31%), enthesopathy (34%) (sites not reported for either), or genu varum (29%). Half (51%) had a history of tooth abscess. A fifth of patients (21%) had experienced fractures in their lifetime, most often in the lower extremities (18%), and 64% had undergone orthopedic surgery, most frequently osteotomy (60%). Two-thirds (64%) of patients had had undergone surgery, this was during childhood for 46% of all patients and as an adult for 27%.

Serum bone biochemistry at baseline and first SF-36 completion is presented in Table 2. Substantial data are missing at SF-36 completion: serum phosphate values were available for 37% of patients, ALP for 24%, PTH for 33%, and 1,25(OH)_2_D_3_ for 14%. The mean serum phosphate concentration at SF-36 completion was 0.65 (SD 0.17) mmol/L (*n* = 45).

**Table 2:** Serum bone biochemistry at baseline and first SF-36 completion.

			**SF-36 completers (*n* = 123)**		**All adults at baseline (*n* = 181)**
			**At baseline**	**At first SF-36 completion**	
**Phosphate**	*n*		106	45	157
	Concentration (mmol/L)	Mean ± SD	0.66 ± 0.17	0.65 ± 0.17	0.66 ± 0.18
	Normality, *n* (%)	Abnormal	88 (83.0)	38 (86.4)^a^	130 (83.3)^b^
		Normal	18 (17.0)	6 (13.6)	26 (16.7)
**ALP**	*n*		68	30	101
	Concentration (U/L)	Mean ± SD	110.9 ± 58.8	117.2 ± 54.7	115.8 ± 57.5
	Normality, *n* (%)	Abnormal	18 (26.5)	9 (30.0)	34 (33.7)
		Normal	50 (73.5)	21 (70.0)	67 (66.3)
**PTH**	*n*		97	41	143
	Concentration (pmol/L)	Mean ± SD	75.1 ± 41.5	81.7 ± 42.6	74.8 ± 44.4
	Normality, *n* (%)	Abnormal	47 (48.5)	22 (53.7)	66 (46.2)
		Normal	50 (51.5)	19 (46.3)	77 (53.8)
**1,25-Dihydoxy vitamin D**	*n*		30	17	50
	Concentration (pmol/L)	Mean ± SD	48.7 ± 22.7	46.6 ± 24.6	48.5 ± 20.6
	Normality, *n* (%)	Abnormal	7 (23.3)	4 (23.5)	11 (22.0)
		Normal	23 (76.7)	13 (76.5)	39 (78.0)

Data for normality of phosphate concentration available for ^a^*n* = 44 and ^b^*n* = 156.

The normality of serum concentrations was determined by the reporting laboratory, according to local reference ranges (which vary between laboratories).

Abbreviations: ALP, alkaline phosphatase; PTH, parathyroid hormone; SF-36, Short-Form 36 Health Survey.

Treatment at baseline and SF-36 completion is reported in Table 3. Overall, 68% of patients who completed the SF-36 were receiving treatment for XLH at baseline, increasing to 81% at SF-36 completion. The proportion receiving treatment with burosumab increased from 18% at baseline to 37% at first SF-36 completion; 50% were taking phosphate and/or active vitamin D at baseline, decreasing to 44% at SF-36 completion (note that patients stopped phosphate/vitamin D when starting burosumab treatment). For those currently taking burosumab, the mean duration of treatment at SF-36 completion was 36.2 (SD 31.5) mo (range 1.1-88.6 mo) (*n* = 48) and the mean (SD) dose was 1.1 (0.2) mg/kg (*n* = 33). The mean (SD) age at the start of burosumab treatment was 42.4 (12.7) yr (*n* = 48) (Table 3). Eleven percent of patients were using opioid pain medication and 36% non-opioid pain medication at first SF-36 completion.

**Table 3:** Treatment for XLH at baseline and first SF-36 completion.

		**SF-36 completers (*n* = 123)**		**All adults at baseline (*n* = 181)**
		**At baseline**	**At first SF-36 completion**	
**Currently receiving treatment, *n* (%)**	Phosphate and/or active vitamin D	62 (50.4)	54 (43.9)	82 (45.3)
	Burosumab	22 (17.9)	46 (37.4)	29 (16.0)
	None	39 (31.7)	23 (18.7)	70 (38.7)
**Previous treatment—all patients, *n* (%)**	Phosphate and/or active vitamin D	109 (88.6)	111 (90.2)	153 (84.5)
	Burosumab	35 (28.5)	48 (39.0)	46 (25.4)
**Previous treatment in patients not currently receiving treatment, *n* (%)**	Phosphate and/or active vitamin D	26 (66.7)	15 (65.2)	43 (61.4)
	Burosumab	11 (28.2)	1 (4.3)	15 (21.4)
**Time on burosumab (mo)**	*n*	35	48	46
	Mean ± SD	29.8 ± 27.0	36.2 ± 31.5	27.7 ± 26.1
	Range	0.0-61.8	1.1-88.6	0.0-61.8
	<1 yr	14 (40.0)	14 (29.2)	19 (41.3)
	1 to <3 yr	5 (14.3)	17 (35.4)	9 (19.6)
	≥3 yr	16 (45.7)	17 (35.4)	18 (39.1)
**Burosumab dose (mg/kg)**	*n*	8	33	12
	Mean ± SD	1.1 ± 0.2	1.1 ± 0.2	1.2 ± 0.3
	Range	0.8-1.4	0.8-1.8	0.8-1.8
**Time on phosphate and/or active vitamin D (mo)**	*n*	108	110	152
	Mean ± SD	193.4 ± 140.2	207.9 ± 146.9	186.9 ± 136.6
	Range	2.6-645.1	3.0-647.2	0.9-645.1
**Age started burosumab (yr)**	*n*	35	48	46
	Mean ± SD	42.1 ± 14.0	42.4 ± 12.7	39.8 ± 15.1
	Range	17.5-69.2	17.5-69.2	15.6-69.2
**Burosumab naïve (yes), *n* (%)**		88 (71.5)	75 (61.0)	135 (74.6)
**Using pain medication at baseline, *n* (%)**	Opioids	9 (7.3)	13 (10.6)	17 (9.4)
	Non-opioids	44 (35.8)	44 (35.8)	52 (28.7)

Abbreviation: SF-36, Short-form 36 Health Survey.

### HRQL

**Figure 1:**
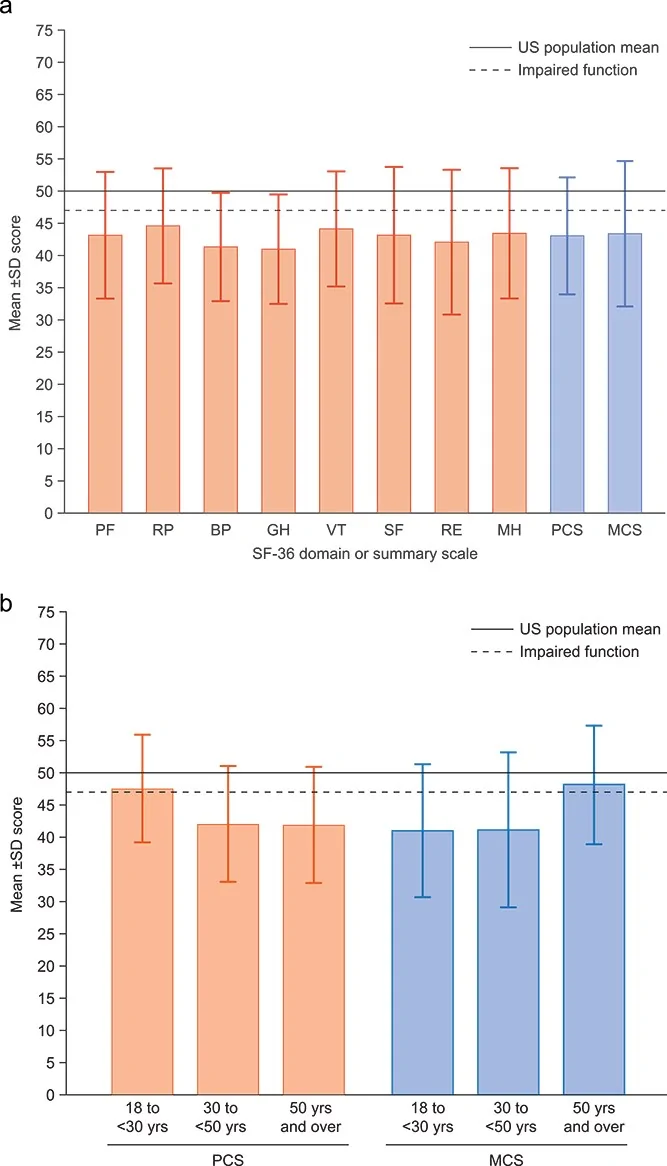
SF-36 scale and summary scores (*n* = 123): (a) All patients, (b) By age group. Each SF-36 scale is scored with the same mean (i.e. 50) and standard deviation (SD) (i.e. 10) found in the 2009 US general population. Group mean scores <47 indicate the impaired functioning on the scale of interest. High scores indicate better HRQL. PF, Physical functioning; RP, Role limitations due to physical health; BP, Bodily pain; GH, General health perceptions; VT, Vitality; SF, Social functioning; RE, Role limitations due to emotional problems; MH, Mental health; PCS, Physical component summary, MCS, Mental component summary.

SF-36 scores at first completion are shown in Figure 1A. Group mean T-scores were <47 on all scale and summary scores, indicating impaired functioning, and were lower (worse HRQL) than the US 2009 general population score of 50.^31^ Mean PCS and MCS scores were similar: 43.0 (SD 9.1) vs 43.4 (11.3). Mean (SD) scale scores were lowest for General health (41.0 [SD 8.5]) and Bodily pain (41.3 [8.4]) and highest for Role physical (44.6 [8.9]) and Vitality (44.1 [8.9]).

Health-related quality of life in different ages groups is shown in Figure 1B. Group mean scores for both PCS and MCS were below the US population mean (worse HRQL) for all 3 age groups (18 to <30, 30 to <50, and ≥50 yr). Only group mean scores for PCS in those aged 18 to <30 yr and MCS in those aged ≥50 yr were slightly above the threshold for impairment. Scores in all other age groups indicated impairment. The younger age group (18 to <30 yr) had greater impairment on the MCS, whereas the older age group (≥50 yr) had greater impairment on the PCS; the middle age group (30 to <50 yr) had impairment on both MCS and PCS.

The proportions of patients reporting no/minimal, moderate, and most detriment in the MCS and PCS items are shown in Figure S1. For all items, there were some patients who reported the greatest level of detriment. The greatest detriment in PCS items was seen in “My health is excellent,” “I am as healthy as anybody I know,” “Vigorous activities, such as running, lifting heavy objects, participating in strenuous sports,” “Bending, kneeling, or stooping,” and “Climbing several flights of stairs.” Greatest detriment in individual MCS items was seen in “Did you feel tired?,” “Did you feel worn out?,” “Did you have a lot of energy?,” “Have you been very nervous?,” and “Have you felt calm and peaceful?”

### Variation in HRQL by demographic, medical history, and treatment characteristics

In terms of demographics, worse physical HRQL (lower PCS score) was significantly related to older age (*r* = −0.194, *p* = .032). In categorical analysis, the lowest mean PCS scores were associated with not being employed (32.5) or being retired/not working due to disability/being a homemaker (37.1) (vs employed/self-employed [44.7], student [55.1], not know/reported [42.8]; *p* < .001) (Table 4). Worse mental health (lower MCS scores) was significantly related to younger age (*r* = 0.231; *p* = .010).

**Table 4:** Associations between SF-36 summary scores and demographics, fracture and surgical history, and XLH treatment (*n* = 123).

**Characteristic**		***N***	**PCS**		**MCS**	
			**Mean or *corr. coeff*.** ^**a**^	***p* value**	**Mean or *corr. coeff*.** ^**a**^	***p* value**
**Demographics and XLH history**						
**Sex**	Female	90	43.7	.193	43.2	.835
	Male	33	41.3		43.7	
**Age at first SF-36 completion (yr)^b^**		123	*−0.194*	**.032**	*0.231*	**.010**
**Age at first SF-36 completion (yr)**	18 to <30	23	47.6	**.027**	41.0	**.005**
	30 to <50	60	42.1		41.1	
	≥50	40	41.9		48.1	
**Employment status**	Student	5	55.1	**<.001**	36.5	.092
	Employed/self employed	51	44.7		40.9	
	Not employed	6	32.5		42.8	
	Retired/disability/homemaker	11	37.1		46.1	
	Not known/reported	50	42.8		46.1	
**Age at diagnosis (yr)^c^**		113	*−0.026*	.788	*0.134*	.156
**Family history of XLH**	Yes	47	44.5	.170	42.5	.653
	No	22	41.1		43.9	
**Lower limb deformities (number of categories)^c^**		123	*0.137*	.131	*0.082*	.366
**Fracture history**						
**Any fracture**	Yes	26	37.4	**<.001**	44.9	.438
	None recorded	97	44.6		43.0	
**Lower extremities**	Yes	22	38.5	**.010**	44.6	.585
	None recorded	101	44.0		43.1	
**Upper extremities**	Yes	5	37.0	.126	48.6	.296
	None recorded	118	43.3		43.2	
**Trunk**	Yes	3	28.9	NA	38.4	NA
	None recorded	120	43.4		43.5	
**Surgical history**						
**Any surgery**	Yes	79	42.1	.105	44.1	.351
	None recorded	44	44.8		42.1	
**Timing—adult**	Yes	33	37.0	**<.001**	44.0	.709
	None recorded	90	45.3		43.1	
**Timing—pediatric**	Yes	57	43.7	.457	45.1	.124
	None recorded	66	42.5		41.9	
**XLH treatment**						
**Current treatment**	Burosumab	46	39.8	**.006**	45.8	.148
	None	23	43.7		43.4	
	P and/or VD	54	45.5		41.3	
**P and/or VD naïve**	Yes	12	44.2	.640	47.1	.225
	No	111	42.9		43.0	
**Time on P and/or VD analogues (mo)^c^**		110	*0.047*	.622	*−0.069*	.641
**Burosumab naive**	Yes	75	45.1	**.002**	42.3	.178
	No	48	39.9		45.1	
**Time on burosumab (mo)^c^**		48	*0.161*	.273	*−0.069*	.641
**Time on burosumab (mo)**	<1 yr	14	37.5	.323	47.9	.110
	1 to <3 yr	17	41.4		40.8	
	≥3 yr	17	40.4		47.1	
**Age started burosumab (yr)^b^**		48	*−0.143*	.323	*0.055*	.703
**Burosumab dose (mg/kg)^c^**		33	*0.191*	.286	*0.076*	.673

a Values are mean SF-36 scores for categorical data (eg, male/female), which were compared using the Student’s *t*-test for two categories or analysis of variance for more than 2 categories.

b Student’s *t*-test/Wilcoxon rank-sum test for 2 categories or one-way/Kruskall–Wallis analysis of variance for more than 2 categories (*in italics*) depending on the normality of the data distribution (*n* ≥ 5 per category required for calculation of *p* values).

c Pearson’s or Spearman’s rank correlation coefficients for continuous data, depending on the normality of the data distribution.

Values are recorded closest to time of first SF-36 completion were used in the analysis.

SF-36 T-Score <47 indicates impaired functioning on the scale of interest. Higher scores indicate better HRQL.

Abbreviations: ALP, alkaline phosphatase; HRQL, health-related quality of life; MCS, Mental health component summary score; NA, not applicable (ie, *n* < 5); P and/or VD, phosphate supplements and/or active vitamin D; PCS, Physical health component summary score; SF-36, Short-form 36 Health Survey; XLH, X-linked hypophosphatemia.

In terms of medical history, worse lower mean PCS scores were associated with history of fracture (37.4 vs 44.6; *p* < .001), lower-extremity fracture (38.5 vs 44.0; *p* = .010), and having surgery as an adult (37.0 vs 45.3; *p* < .001). In terms of XLH treatment, lower mean PCS scores were associated with current burosumab treatment (39.8 vs 43.7; *p* = .006) and any previous burosumab treatment (39.9 vs 45.1; *p* = .002).

### Comparison of SF-36 scores in XLH with other chronic musculoskeletal diseases in adults

**Figure 2:**
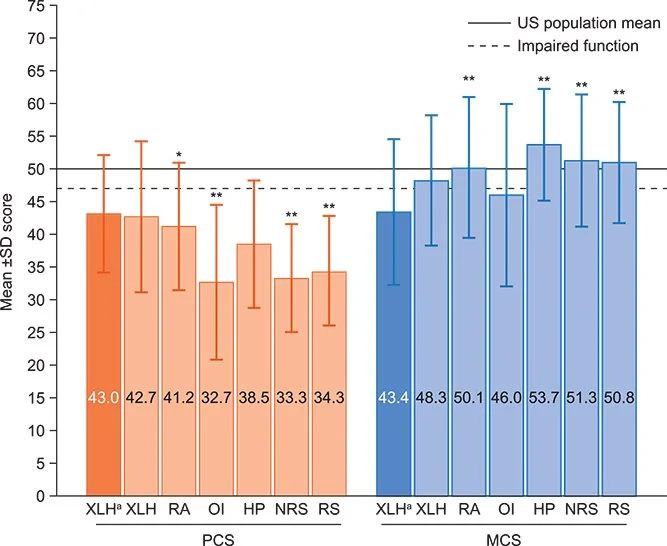
Comparison of SF-36 summary scores in adults with XLH vs other chronic musculoskeletal conditions. Mean scores from current study compared vs published scores using Welch *t*-test based on unequal variance. ^*^p < .05, ^**^  *p* < .01, vs adults with XLH taking burosumab in the current study.^a^ RA, rheumatoid arthritis (*n* = 1884, The Netherlands)^33^; NRS, non-radiographic axial ankylosing spondylitis (*n* = 128; Asia, East and West Europe, and USA)^34^; XLH, X-linked hypophosphatemia, this study (*n* = 46), reported study (*n* = 16, Austria)^32^; OI, osteogenesis imperfecta (*n* = 17, Austria)^32^; HP, hypophosphatasia (*n* = 17, Austria)^32^; and RS, radiographic axial ankylosing spondylitis (*n* = 221, Asia, Europe, and USA).^34^ Details of the populations in the comparator studies are provided in Table S1. Abbreviations: MCS, Mental Component Summary; PCS, Physical Component Summary; SF-36, Short-Form 36 Health Survey; XLH, X-linked hypophosphataemia.

Figure 2 compares SF-36 summary scores (PCS and MCS) from adults in the current International XLH Registry sample, another study of adults with XLH^32^ and published scores for other chronic musculoskeletal conditions; rheumatoid arthritis,^33^ OI, hypophosphatasia,^32^ and radiographic and non-radiographic axial spondyloarthritis.^34^ The patient populations in this comparison are described in Table S1.

PCS and MCS scores in the current study were not significantly different from values reported previously for XLH. For all the comparator conditions, the PCS score was below the mean score for the US population (worse HRQL) and below the threshold for impaired function. Patients with XLH in the current study had significantly higher PCS scores (better HRQL) than adults with rheumatoid arthritis (*n* = 1884, *p* = .038), OI (*n* = 17, *p* < .001), non-radiographic axial spondyloarthritis (*n* = 128, *p* < .001), or radiographic axial spondyloarthritis (*n* = 221, *p* < .001).

MCS scores were lower than the US population mean for XLH (in both the current study and the reported study) and OI. Only the scores for XLH in the current study and OI exceeded the threshold for impaired function. Patients with XLH in the current study had significantly lower MCS scores (worse HRQL) than adults with rheumatoid arthritis (*n* = 1884, *p* < .001), hypophosphatasia (*n* = 17, *p* = .004), non-radiographic axial spondyloarthritis (*n* = 128, *p* < .001), or radiographic axial spondyloarthritis (*n* = 221, *p* < .001).

## Discussion

This analysis explores the HRQL burden in a cohort of French adults with XLH in real-world practice and assesses variation in HRQL according to patient, disease, and treatment characteristics. The analysis demonstrates that HRQL is compromised in patients with XLH, as evidenced by SF-36 scale and summary scores (PCS and MCS) that were lower (worse HRQL) than in the reference US general population and that also exceeded the threshold for impairment (Figure 1A). PCS and MCS scores were similar in this analysis, indicating that this population of adults with XLH experienced mental health burden in addition to physical health burden. This is the first study to report item-level SF-36 data for patients with XLH in the real-world setting; the items with the greatest burden related to overall health and physical activity, energy/fatigue/feeling worn out, and nervousness.

To the best of our knowledge, this is also the first study to report specific factors that are related to variation in HRQL in adults with XLH (Table 4). We found that adults receiving treatment with burosumab had lower PCS scores (worse HRQL) than those receiving phosphate supplements and/or active vitamin D. Similar proportions of patients were receiving burosumab vs phosphate supplements and/or active vitamin D at the time of SF-36 completion. In France, burosumab reimbursement is only for the treatment of severe XLH (ie, refractory to phosphate supplements and/or active vitamin D; complicated severe disease in adolescents with radiographic evidence of bone disease in whom bone growth is complete; and for patients of any age who started burosumab treatment during bone growth and for whom treatment needs to be continued).^35^ Thus, patients receiving burosumab were more likely to have more severe disease than those receiving phosphate supplements and/or active vitamin D or no treatment, which might explain the lower PCS scores. Similarly, an analysis of US patients with XLH found that the burosumab cohort exhibited had a greater disease burden than those taking oral phosphate/active vitamin D.^36^ The most recent European treatment guidelines suggest burosumab treatment for adults with pseudofractures or insufficient musculoskeletal response to oral phosphate and active vitamin D and in symptomatic patients who do not tolerate this treatment or experience toxicity.^23^

Multiple other factors were associated with worse PCS scores: older age, not being employed, history of fracture, lower extremity fracture, and having orthopedic surgery as an adult. Worse MCS scores were associated with younger age. Cheung and colleagues reported that clinical signs and symptoms compromised physical and emotional wellbeing in adults with XLH. Pain and skeletal pathology were the symptoms most frequently reported as interfering most in their lives, as well as complications/pain due to surgery.^13^ A real-world study of adults with XLH found that levels of full-time employment were low in those of working age with XLH, suggesting that XLH has a substantial impact on work productivity; in that study, worse physical function and a greater number of orthopedic surgeries were associated with lower work productivity.^15^

The HRQL of adults with XLH was also compared with that of adults with common chronic musculoskeletal conditions that are comparable to XLH because they begin in childhood, affect the bone, or present with radiological manifestations in early adulthood. Adults with XLH had better physical wellbeing (higher PCS scores) than adults with rheumatoid arthritis, OI, non-radiographic axial spondyloarthritis, or radiographic spondyloarthritis but worse mental wellbeing (lower MCS scores) than those with rheumatoid arthritis, hypophosphatasia, non-radiographic spondyloarthritis, or radiographic spondyloarthritis.

Few studies have reported the HRQL of adults with XLH in real-world settings, and sample sizes in those studies have been small (eg, <50), reflecting XLH as a rare disease. The completeness of the HRQL data in the current real-world study should be highlighted: SF-36 data were available from 123 of 181 adults enrolled in the Registry at French centers—the largest reported HRQL data from XLH patients in the real-world setting to date. This dataset has enabled a more in-depth exploration of HRQL in adult XLH than has previously been possible, with reporting of HRQL for different age groups and exploration of variation in HRQL by patient, XLH, and treatment characteristics. The profile of HRQL in patients with XLH in this registry study differs slightly from that in other real-world settings measured using the SF-36 v2. Whereas we identified similar detriment on both the PCS (mean score 43.0) and MCS (43.4), others report detriment on the PCS but not the MCS.^32^^,^^34^ In a Danish study, adults with XLH (*n* = 49) scored significantly lower than controls on the PCS but not the MCS; 37% were taking phosphate supplements and 53% were receiving active vitamin D but none were being treated with burosumab.^11^ A study of 16 adults with XLH in Austria reported mean PCS and MCS scores of 42.7 and 48.3, respectively, but does not state whether patients were receiving treatment for XLH.^32^ Other studies^16^^,^^18^ used an earlier version of the SF-36 that has slightly different items and a different scoring method^37^ so results are not comparable to the SF-36v2 data in the current study.

The HRQL profile in adults described in this analysis contrasts with that of 96 French children in the International XLH Registry, measured using age-appropriate versions of the Pediatric Quality of Life Inventory.^38^ Whereas adults had similar detriments in both psychosocial and physical aspects of HRQL, children had greater detriment in psychosocial aspects. However, the proportions of patients receiving burosumab treatment differed markedly between the 2 studies: 82% of children compared with 37% of adults. In the children, HRQL was better in those taking burosumab whereas in adults those taking burosumab had worse HRQL—likely reflecting the restricted reimbursement in adults to those with severe disease, explained above, whereas reimbursement for children was not restricted. Children with XLH taking burosumab had better HRQL than children with other chronic musculoskeletal disorders, whereas the adults with XLH taking burosumab in the current study had better MCS but worse PCS than adults with other musculoskeletal conditions.

While real-world studies can provide important insights into disease burden and the effects of treatment outside of the rigorous clinical trial setting, this methodology has limitations in terms of data collection and availability, because data are collected based on routine care and collection is not mandated by the Registry protocol. SF-36 completion was added as a protocol amendment 14 mo after the Registry started collecting data, and completion was recommended but optional. Thus, for some patients, demographic data recorded at baseline (eg, employment status, height/weight/BMI, and family history of XLH) were compared with SF-36 data recorded at a later date (mean 18.6 mo [range 0.1-46.8] after baseline) and may have changed since baseline, although values were not expected to have changed notably for most adults in the analysis. In addition, serum bone biochemistry data at SF-36 completion were limited, because this is not measured routinely in patients with XLH in real-world clinical practice. However, baseline bone biochemistry data cannot be used for comparison with SF-36 values because the interval from baseline to SF-36 completion was too long and would likely have introduced bias, especially given the change in treatment between these two timepoints (eg, 18% on burosumab at baseline vs 37% at SF-36 completion). Other data were also relatively incomplete, such as family history of XLH and burosumab dose. Nevertheless, this is the largest study of HRQL in adults with XLH to date. The SF-36 is a widely used generic measure of HRQL, allowing comparison with other conditions, although use of a US sample as the reference population for generating T-scores is a limitation of this study, as is using US samples for the disease-specific comparisons in the absence of available European data. A bespoke instrument specifically for XLH might enable a more nuanced exploration of disease aspects that most affect patients’ HRQL.

Cross-sectional research has limitations in that it cannot be used to establish cause-effect relationships or to analyze change over time.^39^ While it may be susceptible to recall bias, this was not an issue in the current study because medical history data were captured retrospectively from patients medical records. Nevertheless, cross-sectional analysis is useful to identify potential relationships that can subsequently be evaluated in longitudinal analysis to determine causality. For example, the impact of continued burosumab treatment on HRQL in the real-world setting could be explored in longitudinal analysis. As data in the International XLH Registry accumulate, the impact of burosumab treatment in patients who started treatment at a young age can be explored and compared with patients who started treatment later in the disease course. The bivariate analysis involved testing multiple variables of interest without correction, which increases the likelihood of a type 1 error. The results should therefore be considered exploratory, and contributing to hypothesis development for a future multi-country analysis that will include evaluation of variation in HRQL using multivariable models. The International XLH Registry will also enable a longitudinal study of the impact of burosumab treatment on HRQL.

As a single country study, the generalizability of the findings beyond France may be limited. However, the International XLH Registry is a multi-country study and combining data from all countries may support a future analysis using other statistical methods (eg, multivariable analysis), allowing the impact of treatment on specific aspects of HRQL to be explored. The relationship between burosumab treatment and HRQL in countries with differing reimbursed indications can also be compared when sufficient data have accumulated.

In conclusion, HRQL was impaired across all scales and summary scores of the SF-36 in adults with XLH in France, indicating that these adults with XLH experienced both physical and mental health burdens. More factors were related to PCS than MCS, including age, not working, fracture history, surgery as an adult, and treatment. Only age was related to MCS. Further exploration of the relationships between modifiable influences on HRQL is warranted, including the impact of earlier intervention with burosumab on physical symptoms, notably fractures, and HRQL.

## Supplementary Material

XLH_Registry_French_SF36_supplementary_SEP25_submission_ziag050

## Data Availability

Data that underlie the results reported in this article may be requested. Kyowa Kirin International will review requests individually to determine whether requests are legitimate, relevant, and meet sound scientific principles, and are within the scope of the participants’ informed consent.
