# Correction to: Health-related quality of life in French adults with X-linked hypophosphatemia: real-world data from the International XLH Registry

**DOI:** 10.1093/jbmrpl/ziag132

**Published:** 2026-08-20

This is a correction to: Karine Briot, Sandrine Lemoine, Bernard Cortet, Paulina Szafors, Nadia Mehsen, Guillaume Couture, Kerry Sandilands, Angela J Rylands, Haruka Ishii, Annabel Bowden, Jennifer E Dent, Peter Kamenicky, Health-related quality of life in French adults with X-linked hypophosphatemia: real-world data from the International XLH Registry, *JBMR Plus*, Volume 10, Issue 5, May 2026, ziag050, https://doi.org/10.1093/jbmrpl/ziag050

The following changes have been made to the originally published paper.

Figure 1 has had panel a added, and the initially published Figure 1 now labelled panel b. The caption is corrected so that Figure 1 now reads:


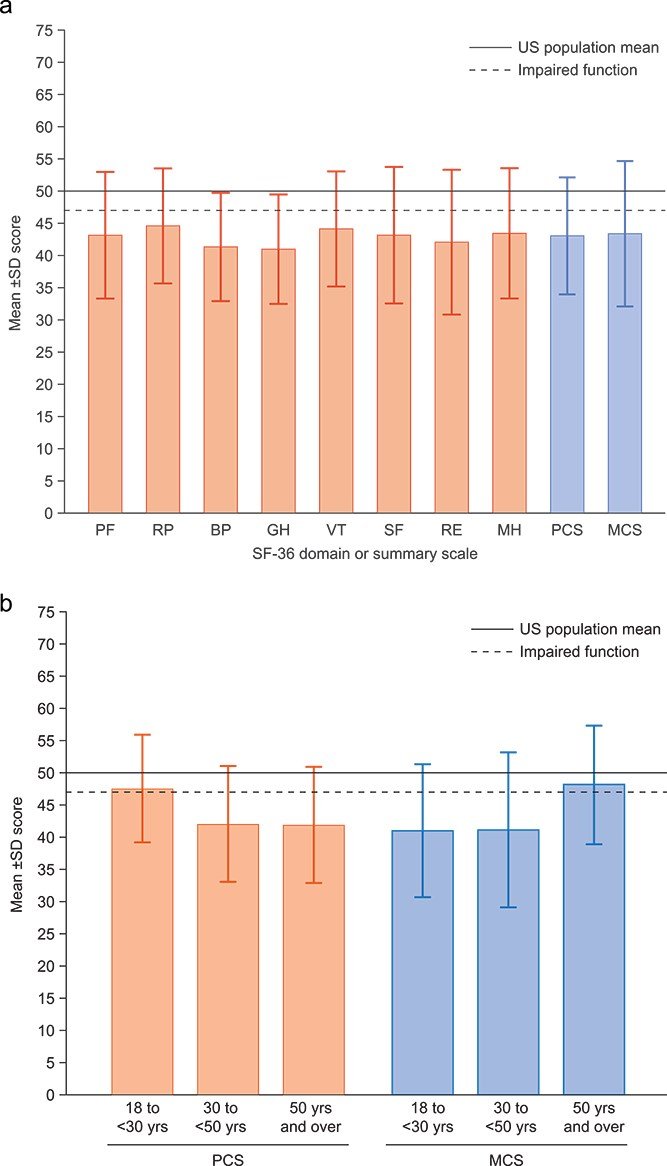


“Figure 1 SF-36 scale and summary scores (n=123)

(a) All patients

(b) By age group

Each SF-36 scale is scored with the same mean (i.e. 50) and standard deviation (SD) (i.e. 10) found in the 2009 US general population. Group mean scores <47 indicate the impaired functioning on the scale of interest. High scores indicate better HRQL. PF, Physical functioning; RP, Role limitations due to physical health; BP, Bodily pain; GH, General health perceptions; VT, Vitality; SF, Social functioning; RE, Role limitations due to emotional problems; MH, Mental health; PCS, Physical component summary, MCS, Mental component summary.”

instead of:


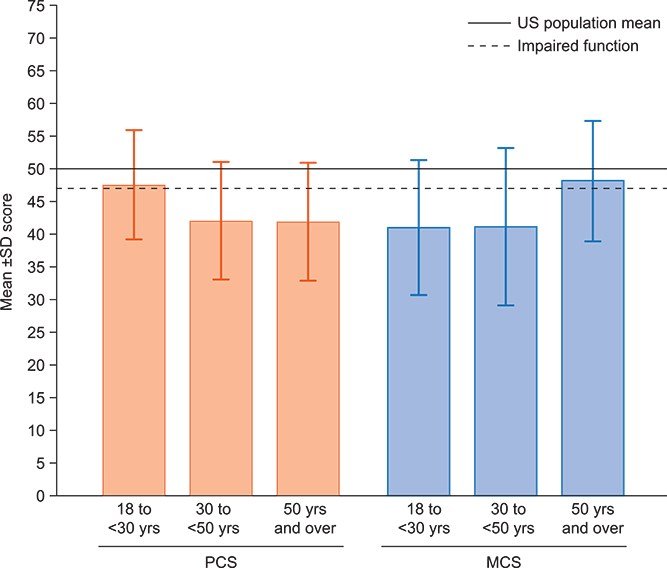


“Figure 1 Short-Form 36 Health Survey (SF-36) scale and summary scores (n=123).EachSF-36scaleisscoredwiththesamemean(ie,50)andSD(ie, 10) found in the 2009US general population. Group mean scores <47 indicate the impaired functioning on the scale of interest. High scores indicate better HRQL. Abbreviations: PF, Physical functioning; RP, Role limitations due to physical health; BP, Bodily pain; GH, General he alth perceptions; VT, Vitality; SF, Social functioning; RE, Role limitations due to emotional problems; MH, Mental health; PCS, Physical Component Summary, MCS, Ment al Component Summary.”

