# Supplementary material for: Health-related quality of life in French adults with X-linked hypophosphatemia: real-world data from the International XLH Registry
Source: JBMR Plus. 2026 Mar 25;10(5):ziag050. doi: 10.1093/jbmrpl/ziag050 (PMC13108448; doi:10.1093/jbmrpl/ziag050)
Supplement: XLH_Registry_French_SF36_supplementary_SEP25_submission_ziag050 [file xlh_registry_french_sf36_supplementary_sep25_submission_ziag050.docx]

Supplementary information

Supplementary Figure 1 SF-36 PCS and MCS item responses (n=123)

(a) PCS


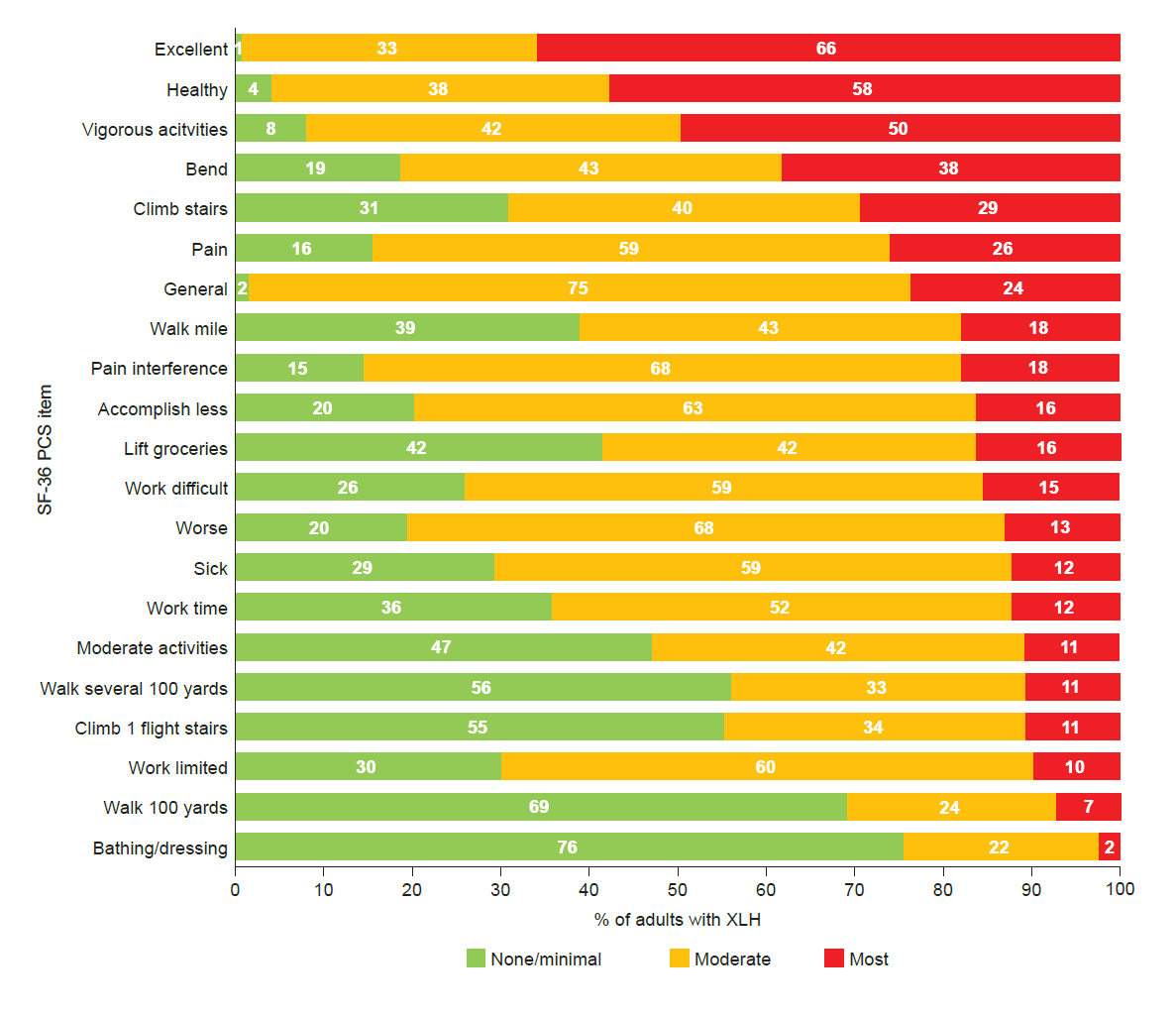


(b) MCS


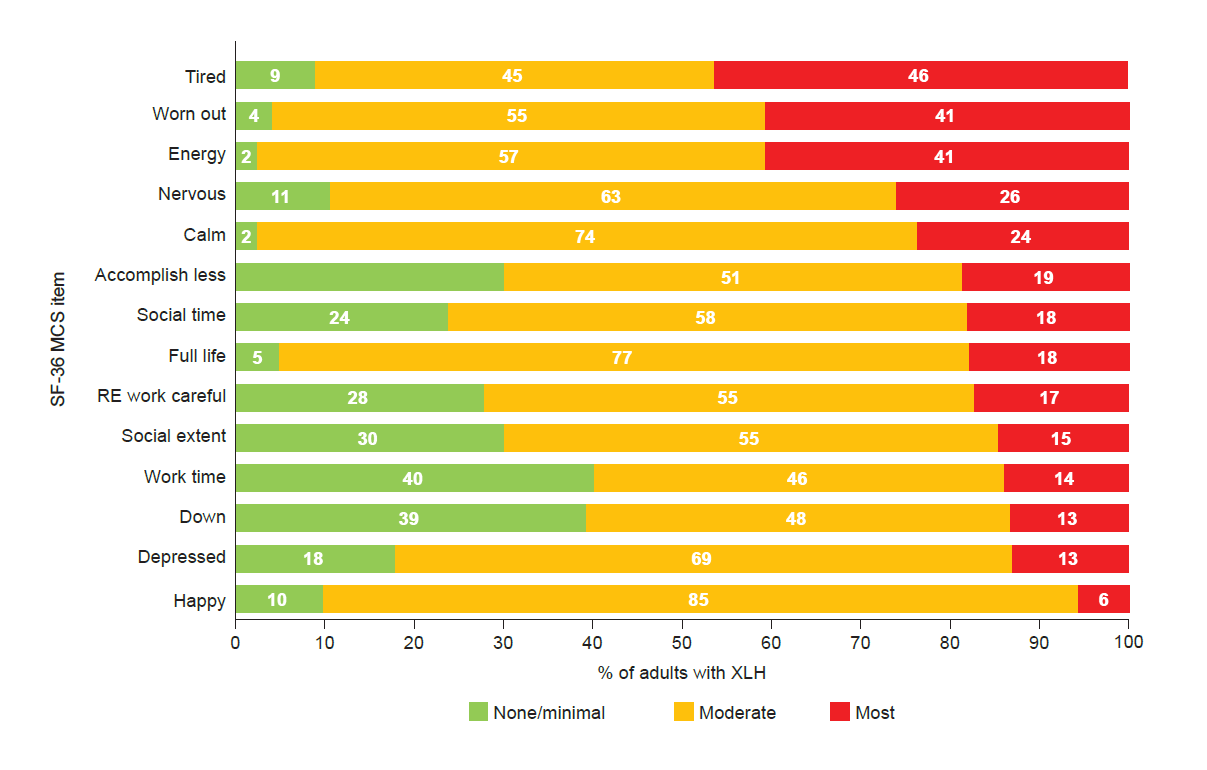


SF-36 items have 3–6 response options; response options have been combined to create three categories to allow comparison across items. Values in bars are percentages of adults with XLH.

PCS abbreviated item content from top to bottom (scale): Excellent = My health is excellent (GH); Healthy = As healthy as anybody I know (GH); Vigorous activities = Vigorous activities, such as running, lifting heavy objects, participating in strenuous sports (PF); Bend = Bending, kneeling or stooping (PF); Climb stairs = Climbing several flights of stairs (PF); Pain = Magnitude of bodily pain (BP); General = Health in general (GH); Walk mile = Walk more than a mile (PF); Pain interference = Extent pain interfered with normal work (BP); Accomplish less = Accomplished less than would have liked; Lift groceries = Lifting or carrying groceries (PF); Work difficult = Had difficulty performing work or other activities (e.g., it took extra effort) (RP); Worse = Expect my health to get worse (GH); Sick = Seem to get sick a little easier than other people (GH); Work time = Cut down the amount of time spent on work or other activities (RP); Moderate activities = Moderate activities, such as moving a table, pushing a vacuum cleaner, bowling, or playing golf (PF); Walk several hundred yards (PF); Climb 1 flight stairs = Climb one flight of stairs (PF); Work limited = Limited in kind of work or other activities (RP); Walk 100 yards = Walking one hundred yards (PF); Bathing/dressing = Bathing/dressing oneself (PF).

MCA abbreviated item content from top to bottom (scale): Tired = Feel tired (VT); Worn out = Feel worn out (VT); Energy = Have a lot of energy (VT); Nervous = Been very nervous (MH); Calm = Felt calm and peaceful (MH); Accomplish less = Accomplished less than you would like (RE); Social time = Frequency health problems interfered with social activities (SF); Full life = Feel full of life (VT); Work careful = Did work or other activities less carefully than usual (RE); Social extent = Extent health problems interfered with social activities (SF); Work time = Cut down the amount of time spent on work or other activities(RE); Down = Feel so down in the dumps that nothing could cheer you up (MH); Depressed = Feel downhearted and depressed (MH); Happy = Been happy (MH).

BP, Bodily pain; GH, General health perceptions; MCS, Mental component summary; MH, Mental health; PCS, Physical component summary; PF, Physical functioning; RE, Role limitations due to emotional problems; RP, Role limitations due to physical health; SF, Social functioning; SF-36, Short-form 36 Health Survey; VT, Vitality; XLH, X-linked hypophosphatemia

Supplementary Table 1 Demographics and clinical history of populations used in comparison of SF-36 scores in Figure 2

| Condition and reference | | | n | | Female (%) | Age (years), mean (SD) | | Geography | | | Treatment at time of SF-36 completion | |  |
| --- | --- | --- | --- | --- | --- | --- | --- | --- | --- | --- | --- | --- | --- |
|  |  |  |  |  |  |  |  | **Country** | **n (%)** | |  |  |  |
| Rare bone diseases ^32^ | Osteogenesis imperfecta | 17 | | 74 | | 48.8 (15.9) | Austria | | 50 (100) | Not reported | |  |  |
|  | Hypophosphatasia | 17 | |  |  |  |  |  |  |  |  |  |  |
|  | XLH | 16 | |  |  |  |  |  |  |  |  |  |  |
| Axial spondyloarthritis ^34^ | Non-radiographic | 128 | | 43 | | 39.5 (11.1) | Asia^a^ | | 15 (11.7) | Bimekizumab (interleukin-17 inhibitor) | |  |  |
|  |  |  |  |  |  |  | E Europe^b^ | | 73 (57.0) |  |  |  |  |
|  |  |  |  |  |  |  | W Europe^c^ | | 31 (24.2) |  |  |  |  |
|  |  |  |  |  |  |  | USA | | 9 (7.0) |  |  |  |  |
|  | Radiographic | 221 | | 28 | | 41.0 (12.1) | Asia^a^ | | 40 (18.1) |  |  |  |  |
|  |  |  |  |  |  |  | E Europe^b^ | | 108 (48.9) |  |  |  |  |
|  |  |  |  |  |  |  | W Europe^c^ | | 67 (30.3) |  |  |  |  |
|  |  |  |  |  |  |  | USA | | 6 (2.7) |  |  |  |  |
| Rheumatoid arthritis ^33^ | | | 1884 | | 64 | 58.3 (13.2) | | Netherlands | 1884 (100) | | Not reported | |  |
| ^a^Turkey, Japan, China  ^b^Bulgaria, Czech Republic, Hungary, Poland  ^c^Belgium, France, Germany, Netherlands, Spain, UK | | | | | | | | | | | | | |
